# Serum Concentrations of AMH and E2 and Ovarian and Uterine Traits in Gilts

**DOI:** 10.3390/ani9100811

**Published:** 2019-10-15

**Authors:** Alicia Steel, Rebecca Z. Athorn, Christopher G. Grupen

**Affiliations:** 1 Sydney School of Veterinary Science, Faculty of Science, The University of Sydney, Camden, NSW 2570, Australia; alicia.steel@sydney.edu.au; 2 Australian Pork Limited, Barton, ACT 2600, Australia; rebecca.athorn@australianpork.com.au

**Keywords:** gilts, gilt selection, sow retention, AMH, E2, ovarian reserve, uterine capacity

## Abstract

**Simple Summary:**

Poor sow retention is a common issue amongst piggeries that creates excessive wastage. Premature culling is largely attributed to reproductive inadequacy. Thus, it is clear the traditional methods for selecting breeding females are inefficient and early markers of reproductive success are required. The aim of this study was to examine whether circulating levels of anti-Müllerian hormone and oestradiol could be useful indicators of ovarian and uterine traits in young gilts. The findings suggest that at a young age, anti-Müllerian hormone could be used to mark the ovarian reserve. Further research into whether the two hormones could be used to mark uterine capacity is needed.

**Abstract:**

Poor sow retention due to reproductive failure is a common reproductive inefficiency amongst piggeries. This shows that traditional methods of gilt selection are inadequate and a marker of reproductive success is needed. The aim of this study was to determine whether circulating levels of AMH and E2 at D80 and D160 are associated with uterine and ovarian traits at D160. Uterine weight, horn length and horn diameter were measured, and ovarian follicle counts were determined histologically. There was a negative relationship between both D80 and D160 AMH levels and D160 ovarian follicle populations. There was also a positive relationship between D80 E2 levels and uterine capacity in gilts that were pubertal at D160. The findings indicate that D80 and D160 AMH could be used to predict ovarian reserve and that D80 E2 levels may be indicative of uterine capacity in precocial gilts.

## 1. Introduction

Globally, poor sow retention is a common reproductive inefficiency amongst piggeries. In Australia, it is estimated that around 40% of sows are culled prior to parity three [1,2,3]. This premature culling has resulted in an average herd parity of just 2.7 [3]. This is concerning considering gilts only become profitable between parities three to six [4]. This is attributed to younger sows having lower pregnancy rates, smaller litter sizes, higher chances of savaging and greater non-productive days [5], as well as to the higher costs required to rear piglets bred from young females [6]. Increasing the average herd parity by a single unit has been shown to be equivalent to a 0.5% increase in lean pork percentage at slaughter [4] In other words, increasing sow retention rates would decrease the input required per kilogram of lean pork. Not only would this be in the best interest of pork producers from an economic standpoint, but also from an efficiency, sustainability and welfare perspective.

It has been known for some time that, in Australian piggeries, the most significant factor contributing to the premature culling of replacement breeding females is reproductive failure [1]. However, the reproductive traits that are considered at gilt selection typically remain limited to teat number, body conformation and dam performance. Selection normally occurs at around 160 days of age and it is not until well after entry into the breeding herd that a gilt’s reproductive potential becomes more evident. Maintaining unproductive individuals up to this point is costly. Hence, an early-age predictive marker for reproductive success is required to aid with the gilt selection process.

Mammals are born with a limited number of ovarian follicles. The number of dormant primordial follicles, which constitutes ovarian reserve, is an important determinant of reproductive potential in females [7]. Obtaining counts of these microscopic follicles is relatively difficult. However, counts of more mature, growing follicle populations can reflect ovarian reserve. This is due to the ovary recruiting primordial follicles to grow in cohorts. The size of these cohorts, at particular stages of development, is proportionate to the pool of primordial follicles the ovary has available to recruit from. The fleets of follicles may not remain proportionate to ovarian reserve at all stages of development due to the differing rates of apoptosis of follicles along the way. Warren et al. [8] has shown that in pigs, the number of growing follicles at intermediate and primary stages are correlated with ovarian reserve, but populations of follicles at later stages are not.

Reproductive potential is also associated with antral follicle populations within ovaries. In young cycling cattle, antral follicle counts (AFC) have been directly linked to the number of morphologically healthy oocytes [9]. Further, AFC is a good marker of ovarian response to exogenous gonadotrophins in assisted reproductive technology cycles [10]. 

In species other than the pig, the circulating level of anti-Müllerian hormone (AMH) has been shown to be a good indicator of ovarian reserve and AFC (cattle [11,12]; sheep [13]; mares [14]; goats [15]; humans [10]; mice [16]). This may be attributed to antral follicles being the site of maximal AMH production and AFC being proportionate to primordial follicle populations in these species. The relationship between circulating AMH and follicle populations in pigs is yet to be distinguished. There is some evidence that indicates AMH may have a unique role in pigs. Expression commences in recruited primordial follicles and, in most species, is maximal at the antral stage before diminishing. However, in pigs, AMH continues to be expressed at similar levels to the antral stages and intensifies in preovulatory follicles [17]. 

Circulating oestradiol (E2) is another candidate hormone that could be indicative of gilts with high reproductive potential. We previously demonstrated that circulating E2 levels in juvenile gilts aged 60, 80 or 100 days of age were positively associated with the probability of stillbirth and negatively associated with the number of piglets born alive in parities one to three [18]. This period was chosen because this is a critical window for ovarian development in pigs [19] and the levels at 80 days of age were found to be the most useful measure. In humans, girls who experience sexual maturity early appear to have lower serum E2 levels than normal-maturing girls when compared at the same sexual developmental stage [20]. Considering the findings of Bidlingmaier et al. [20] and given that E2 exerts a negative-feedback effect on the hypothalamic-pituitary-gonadal (HPG) axis prior to sexual maturity, it may be that the lower E2 levels in juveniles promote earlier establishment of the HPG axis. In gilts, this early development results in regular cycling patterns being established earlier, leading to an increased number of piglets born alive, and higher retention rates to parity three [21,22]. Furthermore, uterine traits such as weight and horn length in gilts aged 160 days of age have been shown to be linked with uterine capacity [23,24] and there is evidence that increasing uterine capacity alleviates issues associated with overcrowding, including early foetal losses, low birth weights and reduced litter size [25]. Thus, we hypothesized that the levels of ovarian hormones in juvenile gilts are related to their ovarian and uterine properties. 

The aim of this study was to measure circulating levels of AMH and E2 in young gilts at 80 and 160 days of age and examine their relationship with ovarian and uterine traits at the age that selection normally occurs. 

## 2. Materials and Methods 

### 2.1. Animals and Ethics 

All animal procedures were conducted with prior institutional ethical approval under the requirements of the NSW Prevention of Cruelty to Animals Act 1985, in accordance with the National Health and Medical Research Council/Commonwealth Scientific and Industrial Research Organisation/Australian Animal Commission’s Code of Practice for the Care and Use of Animals for Scientific Purposes. 

Fifty-four multiplier gilts (F1: Large White x Landrace, PrimeGro^TM^ Genetics, Corowa, NSW) aged 80 days were used in this study. There were five pairs, two trios and one quartet that originated from the same litter. 

From weaning at 28 days of age up until 70 days of age gilts were housed in conventional weaner pens in groups of 45. Gilts were moved to large commercial grower pens and housed on concrete slatted floors in groups of 200 until they were around 130 days old. Gilts were then re-grouped into pens of 50 and housed in conventional finisher pens on concrete slatted and solid floors (50:50) until around 160 days when gilts were slaughtered. 

The feeding system varied throughout the gilts’ lifetime. From weaning until about 125 days of age, gilts were given ad libitum access to a number of commercial weaner and grower diets. Gilts were then fed a specific gilt developer diet ad libitum until slaughter. 

### 2.2. Blood Samples and Assays

Blood samples were collected over consecutive weeks from two groups of gilts (Group 1: N = 26, Group 2: N = 28) at 80 days of age and again at 160 days of age (±4 days). Blood was collected into serum separator tubes using 18 g × 25 mm vacutainer needles and left to clot for 2 h at room temperature. The tubes were then centrifuged at 1000× *g* for twenty minutes and sera separated and stored at −80 °C for less than two months. Five serum samples at D160 were excluded. 

After thawing, serum samples were diluted 1:2 in PBS, and AMH was quantified using a competitive inhibition ELISA kit (CEA228Po: Cloud-Clone Corp, TX, USA) using monoclonal antibodies specific for porcine AMH. In-house validation of this kit was performed as described previously [18]. The minimum detectable dose for the assay kit was 135.8 pg/mL. The intra- and inter-assay precision was <11.4% and <12.9%, respectively.

Oestradiol was measured using a competitive inhibition ELISA kit (CEA461Ge: Cloud-Clone Corp, TX, USA) using a monoclonal antibody specific to E2. The minimum detectable dose for E2 was 46.2 pmol/L and the intra- and inter-assay precision was <2.9% and <14.5%, respectively.

### 2.3. Assessment of Uterine and Ovarian Development

At 160 days of age, gilts were slaughtered at an on-site abattoir. Trimmed carcass weight (CW) and P2 back-fat scores were recorded and reproductive tracts were recovered. The uteri and ovaries from each animal were weighed and the lengths and diameters of each uterine horn were recorded. Uterine diameter measurements were taken at the tubal, middle and cervical ends of each uterine horn. One ovary from each gilt was fixed in 10% neutral buffered formalin for 72 h in preparation for histological analysis. Two uterine measurements were excluded due to damage.

### 2.4. Histological Preparation and Analysis

Ovaries were sliced in half before being embedded in paraffin wax. Starting from the cut in the midline, half of each embedded half-ovary was serially sliced in 5 μm sections using a rotating microtome (Leica^®^, Wetzlar, Germany). Every 40th section was placed in a water bath containing foetal calf serum to aid in fixing the section onto a glass slide. Slides were dried overnight prior to staining with Harris’ Haematoxylin and alcoholic Eosin Y (0.01%). Slide sections were photographed via the Zeiss Axioscan.Z1 at 20× magnification. 

Follicles that contained cross sections of the oocyte were counted and classified by follicle type (primary, secondary, preantral, antral) as described previously [8] with the exception that intermediate follicles were classified as primary follicles. Atretic follicles were not classified by follicle type. Gilts were classified as cycling or non-cycling by the presence of corpora lutea (CL). The total number of follicles was determined according to the methods described by Ireland et al. [9]. For each gilt, total follicle count was calculated by multiplying the total number of follicles for each quarter-ovary by a correction factor of 320 (40 × 4 × 2: 40 accounts for counting every 40th section; four accounts for only slicing one-quarter of the ovary; and two accounts for only testing one ovary per gilt). 

### 2.5. Statistical Analysis

Statistical analysis was conducted using R software version 3.3.3 (R Foundation for Statistical Computing, Vienna, Austria). A significance determination threshold of α = 0.05 was used for all statistical analysis in this study. Replicate (Rep), dam and sire were considered as random factors and were nested to account for genetic and in utero effects. Paired t-tests were performed to assess differences between right and left ovarian and uterine traits. Principal component analysis (PCA) was performed using the *stats::prcomp* function in R to combine uterine weight, length and diameter parameters into principal components (PC) that could best summarise the variation between uteri, and to combine small, primary, secondary, preantral, antral and atretic follicle counts into PCs that best describe the variation in ovarian follicle populations between gilts. Data were scaled prior to PCA. The *gamm::mgcv* function was used to fit multi-dimensional spline models to assess interactions between continuous variables and non-linear relationships. Linear relationships were assessed using *lme4::lmer* and *lme4::glmer* for continuous and binary outcome variables. Carcass weight and P2 fat scores and, where applicable, pubertal status at D160 (as determined by the presence of CL) were considered as predictor variables. Interactions were assessed and removed if insignificant. Missing values were omitted from the analyses. 

## 3. Results

### 3.1. Serum Hormone Levels, Carcass Traits and Ovarian Properties

Table 1 shows a summary of the serum AMH and E2 levels at D80 and D160 as well as carcass, ovarian and uterine measurements at D160 for 54 gilts. Serum levels of AMH increased an average of 8.3% from D80 to D160 (t(47) = −3.17, *p* = 0.003), while E2 levels increased around 11.9% (t(48) = −3.74, *p* < 0.001). Ten gilts were pubertal by D160, as indicated by the presence of CL.

### 3.2. Correlations between Ovarian Hormones and Uterine and Ovarian Traits 

In gilts that were non-cycling at D160, D80 E2 levels were positively correlated with D160 uterine weight and negatively correlated with antral follicle counts. In cycling gilts, there were positive correlations between D160 AMH levels and secondary follicle counts as well as D160 E2 levels and preantral follicle counts (Table 2).

### 3.3. Intra- and Inter-Hormone Correlations

The inter-age and inter-hormone correlations can be found in Table 3. There was a negative correlation between E2 levels taken at different ages, but not between AMH measurements. Further, AMH and E2 levels at D160 were negatively correlated, but this relationship was not observed at D80.

### 3.4. Intra-Uterus Correlations

There were significant correlations between all uterine traits measured (Table 4).

### 3.5. Uterine Mass Indices (UMIs)

**Figure 1:**
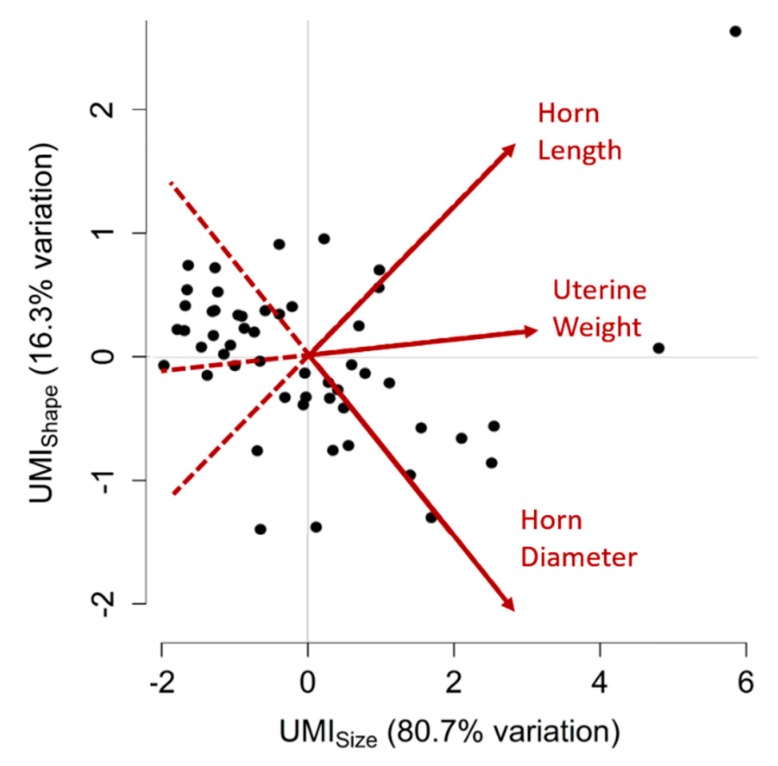
Principal component analysis for uterine traits. Two variables, or Uterine Mass Indices (UMIs), UMI_size_ and UMI_shape_, were created to summarise uterine weight, horn length and horn diameter measurements for each gilt. The two UMIs cumulatively account for 97.0% of the variation observed between uteri. Higher UMI_size_ values correspond to uteri with greater uterine weight, horn length and horn diameter, whereas higher UMI_shape_ values correspond to uteri with greater horn length and uterine weight and lesser horn diameter. Each point on this graph represents a uterus, plotted according to their two UMI values. Points distributed towards the arrowheads of the red vectors have greater measurements of the corresponding uterine trait (labelled in red) than points located towards the tail-end of the vectors. Positively correlated traits have vectors that point in the same direction, negatively correlated traits have vectors that point in opposite directions and traits that have no correlation have vectors that are perpendicular to each other.

Due to the significant intra-uterine trait correlations, PCA was performed. The PCA revealed two PC values that accounted for the significant variation in uterine traits between gilts (80.7% and 16.3%, respectively). The first PC characterises the overall size of the uterus as it is proportionate to uterine weight (loading = 0.62), horn length (loading = 0.57) and horn diameter (loading = 0.54) and is referred to as the Uterine Mass Index of size (UMI_size_). The second PC, referred to as UMI_shape_, describes uterine shape as it is proportionate to horn length (loading = 0.64) and uterine weight (to a weaker extent; loading = 0.08) and strongly inversely proportionate to horn diameter (loading = −0.76). A visual summary of UMI values for each uterus is shown in Figure 1. 

### 3.6. Intra-Ovary Correlations

There were significant correlations between follicle types including between primary and secondary, secondary and atretic, preantral and antral, preantral and atretic and antral and atretic (Table 5).

### 3.7. Ovarian Follicle Indices (OFIs)

**Figure 2:**
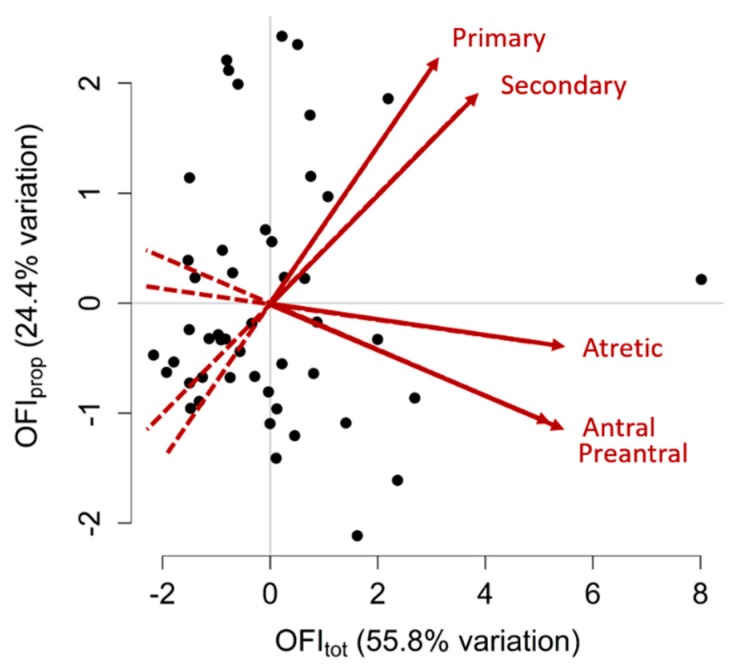
Principal component analysis for ovarian follicle counts. Two variables, or Ovarian Follicle Indices (OFIs), were created to summarise the primary, secondary, preantral, antral and atretic follicle counts of each gilt. This graph shows two OFIs that were created, OFI_tot_ and OFI_prop_, which cumulatively explained 80.2% of the variation in follicle populations observed between gilts. Each point on this graph represents an individual gilt, plotted according to her two OFI values. Gilts with higher OFI_tot_ values have greater follicle numbers of all types, whereas gilts with higher OFI_prop_ values had greater primary and secondary follicle counts and lesser preantral, antral and atretic follicle counts. Points distributed towards the arrowheads of the red vectors have greater counts of the corresponding ovarian follicle type (labelled in red) than points located towards the tail-end of the vectors. Positively correlated traits have vectors that point in the same direction, negatively correlated traits have vectors that point in opposite directions and traits that have no correlation have vectors that are perpendicular to each other.

Due to the significant intra-ovary trait correlations, PCA was performed. The PCA revealed two PC values that accounted for the significant variation in ovarian follicle populations between gilts (55.8% and 24.4%). The first PC characterises the overall follicle populations as it is proportionate to all follicle types measured and is referred to as the Ovarian Follicle Index of total follicles (OFI_tot_). The second PC characterises the ratio of primary and secondary follicles to preantral, antral and atretic follicles and is referred to as OFI_prop_. A visual summary of OFI values for each gilt is shown in Figure 2 and the variable loadings for each OFI can be found in Table 6. There were no significant differences in either OFI values between cycling and non-cycling gilts (*p* > 0.05).

### 3.8. Association between E2 Levels and UMI_size_

**Figure 3:**
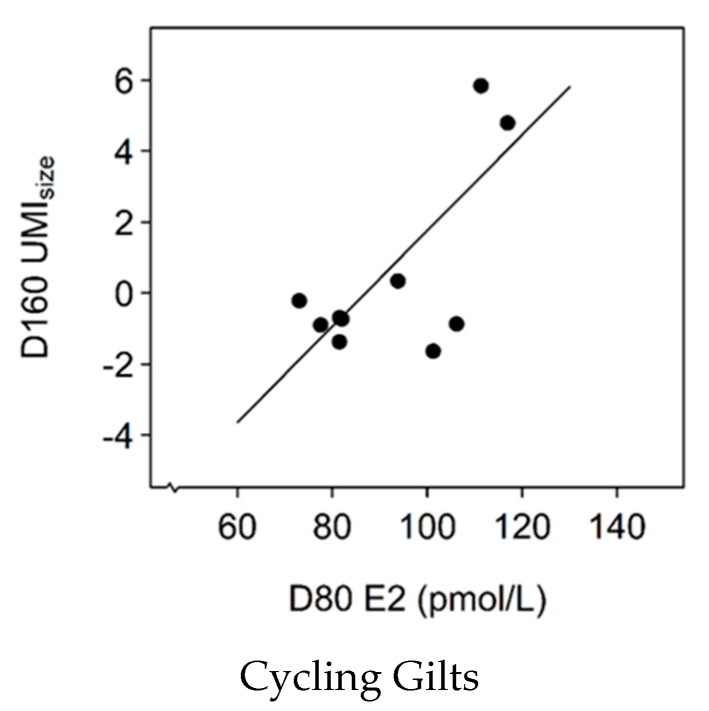
The relationship between serum oestradiol (E2) levels at 80 days of age (D80) and the Uterine Mass Index of size (UMI_size_) in gilts that were cycling at D160 (slope = 0.13, SE = 0.03, χ2(1) = 13.7, *p* < 0.001).

There was a two-way interaction effect between pubertal status and D80 E2 on D160 UMI_size_. There was a positive, linear association between D80 E2 levels and UMI_size_ in cycling gilts at D160 (Figure 3; slope = 0.13, SE = 0.03, χ^2^(1) = 13.7, *p* < 0.001) but there was no relationship between D80 E2 levels and UMI_size_ in gilts that were non-cycling at D160 (*p* > 0.05). 

### 3.9. Association between E2 Levels and OFI_prop_

**Figure 4:**
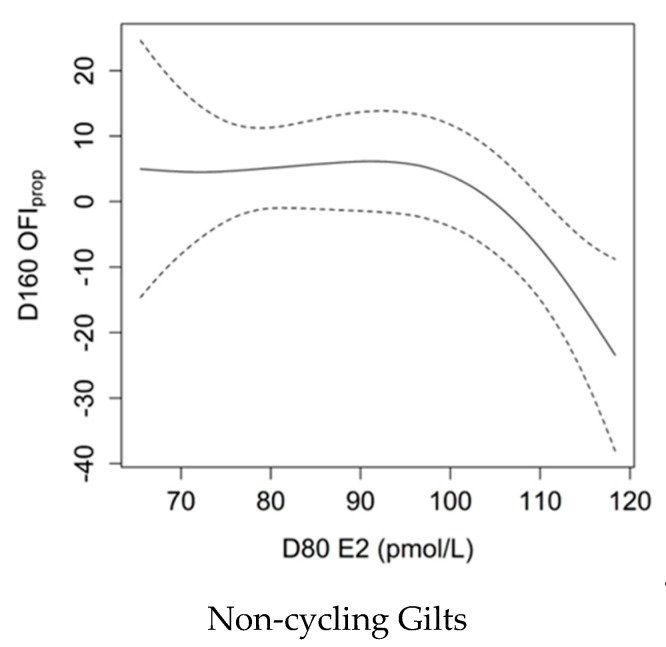
Smoothing spline showing the relationship between serum oestradiol (E2) levels at 80 days of age (D80) and the Ovarian Follicle Index of follicle proportion (OFI_prop_) values in non-cycling gilts at D160 with Bayesian confidence intervals. Greater serum E2 levels corresponded to lower OFIprop values (*p* = 0.027, Adj-R^2^ = 0.14), which represents ovaries with a smaller ratio of primary and secondary follicles to preantral, antral and atretic follicles.

The relationship between E2 at D80 and OFI_prop_ at D160 varied with pubertal status (Figure 4; *p* = 0.027, Adj-R^2^ = 0.14). There was no association between AMH and OFI_prop_ in gilts that had CL present at D160, whereas there was a negative relationship between E2 at D80 and OFI_prop_ in gilts that did not have CL apparent at D160. Such that, non-cycling gilts with elevated D80 E2 (>110 pmol/L) had ovaries with lower primary and secondary follicle counts and greater preantral, antral and atretic follicle counts.

### 3.10. Association between AMH Levels and OFI_tot_

**Figure 5:**
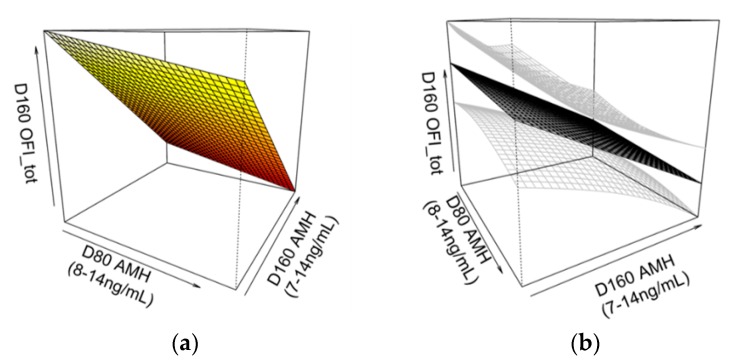
A three-dimensional smoothing spline model showing (**a**) the two-way interaction effect between serum anti-Müllerian hormone (AMH) levels at 80 (D80) and 160 days of age (D160) on OFI_tot_ in non-cycling gilts at D160 (*p* = 0.045, Adj-R^2^ = 0.12). This relationship is also shown (**b**) with standard errors (in grey), rotated for perspective. Pubertal status was determined by the presence of corpora lutea at D160. Non-cycling gilts with both lower D80 and D160 AMH levels had greater OFI_tot_ values. Greater OFI_tot_ values correspond to gilts with greater overall follicle counts (primary, secondary, preantral, antral and atretic).

There was a three-way interaction effect between D80 AMH, D160 AMH and pubertal status on D160 OFI_tot_ (*p* = 0.045, Adj-R^2^ = 0.12). In non-cycling gilts, there was an interacting association between D80 and D160 AMH levels and D160 OFI_tot_ (Figure 5), whereas there was no association in gilts that had CL present at D160. The relationship was such that, the lower the D80 and D160 AMH levels, the greater the overall follicle counts (primary, secondary, preantral, antral and atretic) in gilts that were non-cycling at D160. 

### 3.11. Other Body Condition, Uterine and Ovarian Parameters 

When comparing gilts that were cycling with those that were non-cycling at D160, there were no significant differences in either D80 or D160 ovarian hormone levels, nor were there differences in carcass weight or P2 fat scores (*p* > 0.05). Measurements of serum AMH and E2 at D80 and D160 were not associated with either UMI_shape_, CW or P2 fat scores at D160 (*p* > 0.05). Values of UMI_size_, OFI_tot_ or OFI_prop_ were not associated with CW or P2 fat scores (*p* > 0.05). However, UMI_shape_ varied significantly with P2 fat scores (slope = 0.08, SE = 0.04, χ^2^(1) = 4.32, *p* = 0.038), such that gilts with greater P2 fat scores had heavier uteri with longer, thinner uterine horns. 

## 4. Discussion

This study examined the relationships between ovarian hormones at D80 and D160 and ovarian and uterine traits at D160. Examining this relationship is difficult due to complex ovarian follicle growth dynamics and the significant correlations amongst uterine traits. In order to achieve this, principal component analyses were performed, creating simple summary variables that combined the uterine traits and counts of different follicle types to explain the variation between uteri and ovaries of individual gilts. When considering ovarian follicle populations, most of the variation between gilt ovaries was due to overall follicle counts (as summarised by OFI_tot_), and, to a lesser extent, the ratio of primary and secondary follicles to preantral, antral and atretic follicles (as summarised by OFI_prop_). The ovarian follicle populations measured were independent of carcass weight and fat content. This is consistent with the findings of Schwarz et al. [26] that showed age- and weight-matched gilts have great variation in ovarian activity. There is some evidence that antral follicle populations can vary with growth [27]. However, these measurements were not able to be obtained due to the study being conducted in a commercial production system in which growth rate is not typically measured. Moreover, there were positive correlations between primary and secondary follicle numbers as well as between secondary and atretic and preantral, antral and atretic follicle numbers. A previous study by Warren et al. [8] was similar in that it found pre-antral and antral follicle counts to be positively correlated in porcine ovaries, but differed in that it found no correlation between primary and secondary follicles, and additional positive correlations between secondary and preantral, primary and preantral and primary and antral follicles. 

Results revealed that juvenile (D80) levels of serum E2 were associated with future ovarian follicle populations at D160, the age of selection into the breeding herd. Serum E2 levels at D80 were not linearly correlated with individual follicle counts at D160 in gilts grouped as a whole. However, when grouped according to their pubertal status, there was a non-linear relationship between D80 E2 levels and the ratio of different follicle types at D160 (OFI_prop_) in non-cycling gilts. More specifically, as D80 E2 levels increased over 100 pmol/L, OFI_prop_ values declined, equating to ovaries with smaller proportions of primary and secondary follicles compared to the other, larger (and high-E2 producing) follicle types. Serum concentrations of E2 at D160 were not associated with either OFI, regardless of cycling status.

There were no significant correlations between single measurements of serum AMH concentrations and uterine and ovarian traits when gilts were grouped as a whole, nor was there a correlation between D80 and D160 AMH levels. However, non-linear models showed a two-way interaction between D80 and D160 AMH levels and ovarian follicle populations (OFI_tot_) in non-cycling gilts, such that gilts with lower levels of AMH at both D80 and D160 had greater ovarian follicle populations at D160. The findings suggest that using a combination of both measurements could predict ovarian follicle populations. 

Primordial follicle count (ovarian reserve) was not determined in the present study. Previously, Warren et al. [8] found that the number of primary follicles in pig ovaries was positively correlated with the number of primordial follicles, suggesting that the gilts with lower AMH levels at D80 and D160 (and therefore OFI_tot_) in the present study had a greater ovarian reserve. This negative association contrasts those in other species where AMH has been found to be positively associated with ovarian reserve and antral follicle counts. It should be noted that most livestock studies have examined this relationship in adult females undergoing superovulation cycles, whereas juvenile gilts were examined in the present study. However, though AMH appears to oppose the effects of FSH within the ovary [28], recent studies have found that it stimulates FSH production in the pituitary of prepubertal rats [29]. A negative relationship between AMH levels and HPG development and/or ovarian activity has also been demonstrated in humans as girls with lower AMH at eight years of age are much more likely to have precocial puberty [30]. Thus, the negative relationship between AMH and ovarian follicle populations in this study may be due to AMH having different actions depending on the target tissues and maturational age. 

Both AMH and E2 were found to be associated with ovarian follicle populations but each was associated with a different OFI and, therefore, each represented a different ovarian follicle population dynamic; that is, E2 was associated with the ratio of different follicle types (OFI_prop_) and AMH was associated with overall follicle counts (OFI_tot_). Previously, the porcine ovary has been classified morphologically into two types: The “grape type” with a large number of large surface antral follicles (>5 mm) and atretic follicles and a low number of smaller antral follicles, and the “honeycomb type”, with a large number of small follicles and no large antral follicles [26,31]. When prepubertal ovaries of each type were observed by laparoscopy, after a few days these proportions would change synchronously, and once pubertal, the disparity in follicle numbers between ovary types evened out, showing that porcine ovaries undergo stages of follicular growth in waves [26,31]. Due to the relationship between AMH levels at D80 and D160 and OFI_tot_, the two AMH concentrations determined at D80 and D160 are proposed to be more predictive of ovarian reserve in gilts than the single E2 concentration determined at D80. This could explain why ovarian hormones were only reflective of growing follicle populations in non-cycling gilts and not those that had already begun cycling at D160. 

There was no association between D80 and D160 AMH levels, regardless of pubertal status at D160. A study in sheep also showed no intra-individual correlation of AMH levels between pubertal (3, 4.5 and 6 months) and adult ages (19 months) [32]. The lack of intra-individual correlation of AMH levels may be due to prepubertal AMH peaks being experienced at different ages. Longitudinal studies are required to profile AMH in more detail and determine the maturational age at which AMH would most strongly predict ovarian potential. 

When considering uterine traits, most of the variation in uterine measurements between gilts at D160 was due to differences in overall uterine size (as summarised by UMI_size_) and a small amount of the variation was due to different uterine shape; that is, uteri tended to be either heavier with longer, thinner horns or lighter with shorter, thicker horns (as summarised by UMI_shape_). Though neither UMI values or ovarian hormone levels varied between cycling and non-cycling gilts, pubertal status appeared to affect the relationship between ovarian hormones and uterine traits. That is, there was a positive relationship between D80 E2 levels and UMI_size_ in gilts that were cycling at D160, independent of carcass weight and P2 fat scores. This indicated that gilts with greater D80 E2 levels had greater uterine capacity [23,24]. On the other hand, UMI_shape_ values were independent of ovarian hormone measurements, but varied significantly with P2 fat scores, such that gilts with greater P2 fat scores had heavier uteri with longer, thinner uterine horns. The lack of association between CW and UMI values, which are positively proportionate to uterine weight, does not support previous findings that uterine weight is positively correlated with gilt growth and body weight [33].

## 5. Conclusions

The results showed a relationship between D80 E2 levels and the proportion of primary and secondary follicles in ovaries at D160. There was also a two-way interaction between D80 and D160 AMH levels on overall follicle populations in gilts that were non-cycling at D160, such that the lower the circulating AMH levels at both D80 and D160, the greater the follicle populations. It was deduced that AMH levels at D80 and D160 are predictive of ovarian reserve and that D80 E2 levels are reflective of the follicular waves that occur in gilts prior to puberty. There was a positive association between D80 E2 levels and uterus size in gilts that were pubertal at D160, but the strength of this finding is limited due to the small sample size. Overall, the findings suggest that gilts with lower than average serum levels of AMH at D80 and D160 and E2 levels lower than 100 pmol/L at D80, have greater reproductive potential. Future longitudinal studies are needed to further characterise the relationships observed and to determine whether such hormonal measurements could be effectively applied on-farm to aid in the gilt selection process. 

## Figures and Tables

**Table 1:** Descriptive statistics for serum concentrations of AMH and E2 in gilts at 80 and 160 days of age, and carcass, uterine and ovarian properties at 160 days of age.

	Age (days)	Median	Mean	SD	Range
**Serum hormone levels:**					
AMH (ng/mL)	80	11.1	10.9	1.3	(7.9–13.9)
AMH (ng/mL)	160	12.0	11.8	1.2	(8.9–14.0)
E2 (pmol/L)	80	94.7	94.7	15.7	(65.5–131.0)
E2 (pmol/L)	160	104.2	106.2	14.7	(72.6–143.1)
**Carcass traits:**					
CW (kg)	160	78.0	78.1	10.0	(58.1–112.4)
P2 fat score (mm)	160	13.2	13.4	2.9	(8.4–24.0)
**Uterine traits:**					
Weight (g)	160	77.6	102.0	70.9	(26.7–370.9)
Diameter (mm)	160	12.3	12.3	2.3	(8.6–17.2)
Length (mm)	160	496.3	533.4	176.5	(252.5–1450.0)
**Ovarian follicle counts:**					
Primary	160	49,440	51,543	24,376	(14,000–120,000)
Secondary	160	31,360	37,374	21,636	(9600–104,640)
Preantral	160	3840	5502	5479	(0–29,964)
Antral	160	5600	7004	5430	(640–29,091)
Atretic	160	22,880	28,756	21,970	(1920–107,636)
Total (Healthy)	160	92,840	101,423	44,126	(42,880–275,814)
Total (Healthy + Atretic)	160	112,427	130,179	58,613	(52,160–383,451)

AMH: Anti-Müllerian hormone; E2: Oestradiol, CW: Trimmed carcass weight.

**Table 2:** Pearson’s correlation coefficients between ovarian hormones at 80 and 160 days of age (D80 and D160, respectively) and uterine and ovarian traits at D160.

	D80		D160	
	AMH	E2	AMH	E2
Uterine Weight	−0.25 ^	0.23	0.02	−0.08
Horn Diameter	−0.23	0.04	0.10	0.02
Horn Length	−0.25 ^	0.21	0.17	−0.09
Primary	0.02	0.02	0.21	0.00
Secondary	0.04	−0.18	0.23	−0.03
Preantral	0.00	−0.24 ^	−0.07	0.37 *
Antral	0.02	−0.13	0.08	0.26 ^
Atretic	−0.10	−0.16	0.18	0.11

^ *p* < 0.1 * *p* < 0.05; AMH: Anti-Müllerian hormone; E2: Oestradiol.

**Table 3:** Pearson’s correlation coefficients between ovarian hormones at 80 and 160 days of age (D80 and D160, respectively).

		D80		D160	
		AMH	E2	AMH	E2
**D80**	AMH				
	E2	0.23 ^			
**D160**	AMH	0.13	−0.05		
	E2	−0.07	−0.32 *	−0.29 *	

^ *p* < 0.1 * *p* < 0.05; AMH: Anti-Müllerian hormone; E2: Oestradiol.

**Table 4:** Pearson’s correlation coefficients between uterine traits at 160 days of age.

	Uterine Weight	Horn diameter	Horn Length
Uterine Weight			
Horn diameter	0.76*		
Horn Length	0.84 *	0.52 *	

* *p* < 0.001.

**Table 5:** Pearson’s correlation coefficients between ovarian follicle populations at 160 days of age.

	Primary	Secondary	Preantral	Antral	Atretic
Primary					
Secondary	0.52 *				
Preantral	0.17	0.28 ^			
Antral	0.22	0.23	0.84 *		
Atretic	0.22	0.48 *	0.61 *	0.72 *	

^ *p* < 0.1 * *p* < 0.001.

**Table 6:** Table of variable loadings from the principal component analysis.

Follicle Type	Loading	
	OFI_tot_	OFI_prop_
Primary	0.28	0.66
Secondary	0.36	0.57
Preantral	0.50	−0.33
Antral	0.52	−0.34
Atretic	0.51	−0.10

OFI: Ovarian Follicle Index.
